# A single black ulcer in a child with acute lymphocytic leukemia*

**DOI:** 10.1590/abd1806-4841.20164989

**Published:** 2016

**Authors:** Michelangelo Vestita, Angela Filoni, Nicola Santoro, Gianpaolo Arcamone, Domenico Bonamonte

**Affiliations:** 1 Centro Di Riferimento Oncologico Della Basilicata (IRCCS), Via Padre Pio, Italy; 2 University of Bari – Bari, Italy

**Keywords:** Pseudomonas aeruginosa, Bacillus anthracis, Ulcer

## Abstract

Ecthyma gangrenosum is an uncommon dermatological manifestation characterized by round, indurated ulcers with a central necrotic black eschar and surrounding erythema. This report describes the case of a 5-year-old girl, affected by acute lymphocytic leukemia, presenting with a black eschar on her right thigh. Such lesions should always be correctly identified to avoid potentially fatal bacteraemia. Furthermore, because of its similar clinical presentation, cutaneous anthrax must be ruled out.

## INTRODUCTION

Ecthyma gangrenosum (EG) is an uncommon dermatological manifestation, characterized by round, indurated ulcers with a central necrotic black eschar and surrounding erythema. EG typically presents with multiple lesions, though patients with single lesions have been observed.^1^ Although any cutaneous site can be involved, the gluteal and perineal regions are most commonly affected . ^2^

## CASE REPORT

**Figure 1:**
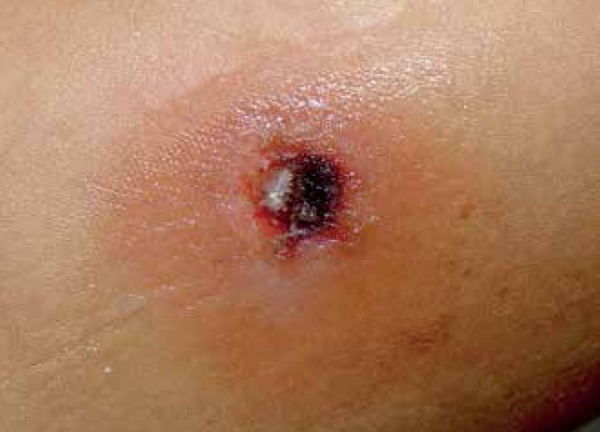
Black central eschar surrounded by an erythematous-edematous halo on the right thigh.

A 5-year-old girl presented with an erythemato-edematous lesion with a black central eschar on the lateral surface of her right thigh (Figure 1).

She had been diagnosed with acute lymphocytic leukemia (ALL) three months before and had been hospitalized 10 days prior to the first observation, in order to commence Phase Ib chemotherapy of the AIEOP-BFM ALL 2009 protocol. ^3^

The cutaneous lesion had allegedly appeared 3 days previously as a painless, pruritic papule, which had progressively enlarged and developed an overlapping vesicle. The latter had later burst centrally, leaving an ulcerative necrotic area, with the subsequent formation of a black eschar, centrifugally surrounded by a vesicular edge, and a circular erythemato-edematous area. The central lesion measured approximately 3cm.

General physical examination only showed reactive right inguinal lymphadenopathy. Moreover, the patient had suffered from intermittent fever at 38C° for a few days before the cutaneous lesion onset. Blood examination only revealed lymphocytosis and mild anemia.

By the time of the first appointment, she was undergoing treatment with ceftriaxon, fluconazole and teicoplanin. The former two were part of the chemotherapy infection-prevention scheme, while the latter had been empirically administered since the day of the cutaneous lesion onset.

Given the peculiar clinical presentation, cutaneous anthrax from *Bacillus anthracis* was immediately suspected. Differential diagnoses included ecthyma, skin infection caused by other bacteria (*Staphylococcus aureus, Pseudomonas aeruginosa*), herpes simplex, and tularaemia. ^4,5^

Two days after lesion onset, a cutaneous, lesional swab resulted. It came back negative for fungal infections and positive for *Pseudomonas Aeruginosa*. As soon as these results came in, intravenous amikacin was added to the patient's therapy.

Blood cultures had been carried out at the onset of fever and on three consecutive days following the cutaneous lesion's appearance; they were all negative.

Four days after the first amikacin administration, the patient showed improvement, with edema and erythema resolution. IgM antibodies against *Francisella Tularensis* and HSV1-2 were negative.

The lesion continued to heal slowly over the following weeks.

## DISCUSSION

EG is usually associated with life-threatening, systemic infection by *Pseudomonas aeruginosa* in immunocompromised individuals, frequently linked to neutropenia or malignancy.^2^ However, EG rarely develops due to *Pseudomonas aeruginosa* in the absence of bacteraemia. ^6-8^ In the latter case, patients tend to have a better prognosis, with a mortality rate of 16% compared with 38-96% for individuals with bacteremia. ^1^

The pathogenic mechanism in the development of EG remains unknown. Nonetheless, nonbacteremic EG is thought to follow direct inoculation of the organism in a prior site of trauma.^1,9^ In this case, the patient had no recollection of any previous trauma and had been hospitalized for days before lesion onset. Another explanation may entangle a minor bacteraemia, in which pathogenic blood spread is transient, therefore resulting in a single lesion.

Appropriate therapy implies early recognition of skin lesions and specific antibiotic therapy. Association therapy entailing an anti-pseudomonal β-lactam antibiotic and an aminoglycoside is the recommended regimen for both bacteremic and nonbacteremic *Pseudomonas aeruginosa* EG. ^6,7^

This report described a rare case of EG from *Pseudomonas aeruginosa* in a child with ALL who presented with a single lesion and no associated bacteraemia. Such an atypical presentation of EG should always be considered and promptly identified to avoid potentially fatal bacteraemia in immunocompromised patients. Furthermore, in this case, the clinical appearance led the medical staff to consider and subsequently exclude cutaneous anthrax.^10^ Indeed, the authors advise that *Bacillus anthracis* infection should always be considered in the event of a single, painless, pruritic papule, vesicle or ulcer exhibiting an overlapping black eschar, often with massive surrounding edema.
